# Hartmann’s Reversal: A Single-Centre Experience

**DOI:** 10.7759/cureus.31654

**Published:** 2022-11-18

**Authors:** Sadaf Zehra, Muhammad Khawar Abbas

**Affiliations:** 1 General Surgery, Kettering General Hospital, Kettering, GBR; 2 Medicine and Surgery, Northwick Park Hospital, London, GBR

**Keywords:** colorectal cancer, diverticulitis colon, stoma, colorectal diseases, reversal of hartmann's procedure, hartmann’s procedure

## Abstract

A proctosigmoidectomy, commonly called Hartmann’s procedure (HP), is the surgical resection of the rectosigmoid colon with the closure of the anorectal stump and creation of an artificial stomal opening (ostomy) on the abdomen (colostomy). It is generally performed with the intention of reversal once the underlying cause is treated. The aim of this study is to assess the predictive factors and intra-operative difficulties that might influence the decision to indicate or contra-indicate stomal reversal after HP. Patients who underwent HP between January 2010 and December 2017 were retrospectively evaluated in a single institution. Preoperative, intraoperative, and postoperative data were analysed for patients who underwent HP for benign as well as malignant conditions. The reversal rate was comparable with the proportion of benign cases, consistent with published evidence that reversal rates for diverticular disease are higher as compared to colorectal cancer. Disease progression/metastasis, advanced age, multiple co-morbidities, and procedure abandonment (frozen pelvis /leak) were the most common contra-indications for reversal.

## Introduction

A proctosigmoidectomy, commonly called Hartmann’s procedure (HP), is the surgical resection of the rectosigmoid colon with the closure of the anorectal stump and creation of an artificial stomal opening (ostomy) on the abdomen (colostomy). It was originally proposed by Henri Albert Hartmann in 1921 at the 30th Congress of the French Surgical Association. At the congress, Hartmann described the success of his surgical strategy in treating two patients with acute obstructive cancer of the sigmoid colon [1]. The goal of HP is to allow the passage of faeces in patients with acute left colonic complications precipitated by large-bowel obstructive diseases such as diverticulitis, hernia, colorectal cancer, extracolonic cancer (carcinoid, peritoneal carcinomatosis, lymphoma, and gastrointestinal stromal tumour), and colonic volvulus including inflammatory bowel disease [2,3]. It is also increasingly used to treat faecal incontinence in elderly and frail patients [4]. HP has shown better results in terms of significantly decreased morbidity and mortality [5].

While HP is usually performed as a temporary life-saving emergency or elective procedure with the intention of reversing it later on upon successful treatment of the underlying causes of acute large-bowel obstruction, about 20%-50% of HPs are never reversed [5,6]. Restoration of bowel continuity after HP is considered a highly risky procedure in terms of post-reversal complications (especially anastomotic leakage and diarrhoea) and mortality (5%-10%) [7]. However, some studies consider HP reversal safe with acceptable morbidity (16.1%-21%) and mortality rates (0.77%-3.6%) [6,8-10]. The success of HP reversal is highly dependent on a careful selection of patients with respect to less-advanced age, male gender, low pre-operative comorbidities based on the Charlson comorbidity index and non-neoplastic aetiology [9]. Majority of reasons to contraindicate HP reversal are often related to pre-operative comorbidities and the involvement of underlying metastatic disease [7,9,11]. Furthermore, the two surgical techniques used for HP reversal; open Hartmann’s reversal (OHR) and laparoscopic Hartmann's reversal (LHR), have different success rates, with the latter being the most preferred surgical strategy [12,13]. The laparoscopic approach to HP reversal is associated with the benefit of faster recovery time and fewer post-reversal complications as compared to OHR [12]. In addition, the experience of a colorectal surgeon is a significant factor in the success of HP reversal, especially if the laparoscopic approach is chosen [11,13]. Published evidence on the decision to indicate or contraindicate HP reversal is still not clear. Thus, the aim of this study is to assess the predictive factors and intra-operative difficulties that might influence the decision to indicate or contra-indicate stomal reversal after HP in patients with large-bowel obstruction.

## Materials and methods

Patients and procedure

This study was exempted from the Institutional Review Board (IRB) clearance as it was a retrospective study with no direct or indirect identifiers of human data. Patients who underwent elective or emergency HP for the treatment of benign or malignant large-bowel obstruction at a single institution between January 2010 and December 2017 were evaluated retrospectively. Medical charts were reviewed to identify preoperative, intraoperative, and post-operative data. Preoperative data included patients’ age, gender, American Society of Anesthesiologists (ASA) physical status score, BMI, and indications for HP (benign and malignant large-bowel obstruction disease) and subsequent HP reversal. Intraoperative data included the type of surgical technique (laparoscopic versus open procedure) and operative time. Post-operative data included the period between HP and reversal, length of hospital stay, postoperative complications (medical and surgical) and mortality. Post-operative complications were assessed and categorised based on the Clavien-Dindo Classification (CDC) criteria for surgical complications (Table 1) [14,15]. In addition, the risk of postoperative complications was assessed based on the peritoneal adhesion index (PAI), which is a score based on the appearance and distribution of peritoneal adhesions.

**Table 1:** Clavien-Dindo Classification (CDC) criteria for surgical complications

	DEFINITIONS
I	Any deviation from the normal postoperative course without the need for pharmacological treatment other than the “allowed therapeutic regimens”, or surgical, endoscopic and radiological interventions.
II	Requiring pharmacological treatment with drugs beyond those allowed for grade 1 complications. Blood transfusions and total parenteral nutrition are also included.
III	Requiring surgical, endoscopic or radiological intervention.
IV	Life-threatening complication requiring critical care management; CNS complications including brain haemorrhage and ischemic stroke (excluding TIA), sub-arachnoid bleeding.
V	Death of a patient
STARSurgII: DISCOVER Protocol Launch 16^th^ September 2014, Royal College of Surgeons of England	

Statistical analysis

All numerical values were presented as mean and standard deviation (SD), ratios, or percentage proportions. Bivariate analysis was used to compare post-operative complications based on PAI score, where the Mann-Whitney U test was applied for continuous variables and Chi-square or Fisher's exact test for categorical variables. A two-sided p-value of ≤ 0.05 was considered to be significant. Statistical analyses were performed by using IBM SPSS Statistics for Windows, version 19 (IBM Corp., Armonk, NY, USA).

## Results

A total of 89 patients underwent HP for a variety of indications during the 8-year retrospective period. The sample population comprised slightly more males (52.81%) than females with an overall mean (SD) age of 67.3 ± 14.5 years. With regards to physical health, the sample patient population was generally overweight (BMI was 29.2 ± 3.5). However, their preoperative physical status was generally good, ranging from “normal health” to “mild systemic disease” as shown by the mean (median) ASA score of 2 (1). The most frequent indications for HP were diverticulitis (51.68%) and colorectal cancer (39.32%), with others accounting for only 9%. There were more benign (59.6%) than malignant cases of large-bowel obstruction. There were more emergency HPs cases (61.8%) than elective cases.

Restoration of intestinal continuity was attempted in 45 (50.6%) patients, with the mean interval time until reversal being 11.9 ± 8.3 months. OHR was the most preferred surgical technique compared to LHR (36:9) and the mean operative time was 185.8 ± 72.3 minutes. The most and least common reasons for not undergoing HP reversal included disease progression/metastasis (25.1%) and abandoned procedures due to frozen pelvis /leak (6.9%), respectively. The overall PAI score was 13.2 ± 5.3, with significant variations between subgroups of patients with or without post-operative complications or post-operative bowel obstruction (P < 0.001). The PAI score was higher in patients with post-operative complications (18.64 ± 2.68) compared to patients without post-operative complications (10.48 ± 3.91). A similar trend was noted in patients with (19.67 ± 2.18) and without (11.36 ± 4.34) post-operative bowel obstruction. With regards to peritoneal adhesions, strong and flimsy adhesions were the most and least common types, respectively. CDC scores were not reported in the majority of the patients (66.7%); with the majority of the remaining patients (20.0%) having a score of 3. Only a single mortality case (2.2%) was reported. The mean length of hospital stay (LOS) was 8.8 ± 3.8 days (Table 2).

**Table 2:** Patient demographics and results

Patient and clinical data	HP; N = 89
Sex (M : F)	47 : 42
ASA (median)	2 (1)
BMI	29.2 ± 3.5
Emergency : Elective	55 : 34
Benign, n (%)	53 (59.6%)
Malignant, n (%)	36 (40.4%)
No of HR, n (%)	45 (50.6%)
Hartmann’s indication; n (%)	
Diverticulitis	46 (51.68%)
Colorectal cancer	35 (39.32%)
Others	8 (9%)
Interval (months), mean ± SD	11.9 ± 8.3
Laparoscopic: Open reversal	9 : 36
Mean operative time (minutes)	185.8 ± 72.3
Type of adhesions; n (%)	
Flimsy adhesions	1 (2.2%)
Strong adhesions	23 (51.1%)
Very strong adhesions	21 (46.7%)
PAI (Peritoneal adhesion Index) score; mean ± SD	
Post-operative complications	18.64 ± 2.68
No post-operative complications	10.48 ± 3.91
Postop. bowel obstruction	19.67 ± 2.18
No postop. bowel obstruction	11.36 ± 4.34
Overall PAI score	13.2 ± 5.3
Clavian-Dindo Classification score; n (%)	
None	30 (66.7%)
Class 1	2 (4.4%)
Class 2	3 (6.7%)
Class 3	9 (20.0%)
Class 4/5	1 (2.2%)
Reason for HR contraindication; n (%)	
Advance age	9 (20.5%)
Multiple Co-morbid	5(11.4%)
Patient decision	6(13.6%)
Disease progression/metastasis	11(25.0%)
Procedure abandon (frozen pelvis /leak)	3(6.8%)
Length of stay (days) mean ± SD	8.8 ± 3.8
Mortality; n (%)	1 (2.2%)
Morbidity; n (%)	14 (31%)

## Discussion

Treatment of acute large-bowel obstruction due to diverticular disease and colorectal cancer involves primary resection followed by anastomosis. However, this operation is preferably performed as a multistage procedure as a precautionary measure to avert intra- and post-operative risks, especially in emergency cases [10,16]. While HP allows a multistage approach to treating large-bowel obstruction, its reversal rate to allow the completion of anastomosis is still low; usually, 50% as demonstrated in the present study, or lower (< 50%) as demonstrated in published studies by Hallam et al. (47%), Vermeulen et al. (45%) and Roig et al. (32%) [6,11,17]. It appears that making a decision to indicate or contra-indicate the reversal of HP is difficult as it is influenced by various challenging factors including patient choice (or consent) and potential stoma-free length of survival as well as local disease factors [16]. The mean interval between HP and Hartmann's reversal in this study was 11.9 (+/- 8.3) months. This long interval between HP and Hartmann's reversal (HR) can be attributed to the elective nature of the reversal. Human factors including the time taken to make a decision for reversal and the pre-operative investigations were also contributing factors. However, the interval between HP and HR was noted to vary widely in previous studies with one study showing a mean reversal interval of 9.1 (0.4-25.8) months and another showing a median of 11 (4-96) months [6,11].

In the present study, around half of the indications of HP were diverticulitis. While this is inconsistent with numerous other studies, which have shown colorectal cancer as the most common indication for HP [10], more than half of the patients in the present study had a benign obstructive large-bowel disease. The reversal rate was comparable with the proportion of benign cases, consistent with published evidence that reversal rates for the diverticular disease are higher (31%-85%), as compared to 4%-53% for colorectal cancer [6,16]. Clearly, it appears that the reversal of HP for colorectal carcinomas presents a higher risk and lower stoma-free survival period compared to reversal involving benign disease, such as diverticulitis as is the case with the current study supported by previous studies [6].

LHR is the most preferred approach, which is associated with faster recovery time and fewer post-reversal complications as compared to OHR [12,13,18]. However, OHR was the most preferred approach in the present study, where a considerably low mortality rate (only one case) was recorded.

Careful selection of suitable patient candidates for reversal has a significant influence on success rates. In the present study, disease progression/metastasis, advanced age, multiple co-morbidities, and procedure abandonment (frozen pelvis /leak) were the most common contra-indications for reversal in descending order. This is consistent with a study by Hess et al., which demonstrated that reversal was less likely in patients with preoperative immunosuppression or chemotherapy and elderly patients [19]. Similarly, the study by Vermeulen et al. showed that comorbidities, age, disease progression, and patients' choices were the most common reasons for not undergoing the reversal [11]. Likewise, other studies have shown that reversal is highly dependent on a careful selection of patients with respect to less-advanced age, male gender, low pre-operative comorbidities based on the Charlson comorbidity index, and non-neoplastic aetiology [9].

The findings of this study should be seen in light of a few limitations including the sample size and limited demographic variability. There is little data available about rehabilitation post-surgery and the provision of community support to the patients as these factors influence the patient's decision regarding the reversal of HP. These factors should be considered in future studies.

## Conclusions

HP is more commonly performed for benign disease rather than colorectal cancer resulting in a low complication rate and acceptable rates of stoma reversal. The finding of age, patient motivation and disease progression have an impact on the decision for reversal. Despite intra-operative difficulties, there is an utmost need to further explore and research the appropriateness of a single-stage procedure with primary anastomosis against a two-staged HP with stoma and reversal. There is also a need to investigate the impact of post-operative rehabilitation and community support on patients' choice and motivation for undergoing the reversal of HP.
